# Fatty acid β-oxidation promotes breast cancer stemness and metastasis via the miRNA-328-3p-CPT1A pathway

**DOI:** 10.1038/s41417-021-00348-y

**Published:** 2021-05-27

**Authors:** Feng Zeng, Mingkang Yao, Yun Wang, Wei Zheng, Shengshan Liu, Zeyu Hou, Xiaoming Cheng, Suhong Sun, Taolang Li, Hongyuan Zhao, Yi Luo, Jiang Li

**Affiliations:** 1 Thyroid and Breast Surgery, Affiliated Hospital of Zunyi Medical University, Zunyi, Guizhou China; 2 Thyroid and Breast Surgery, The second Affiliated Hospital of Zunyi Medical University, Intersection of Xinpu Avenue and Xinlong Avenue in Xinpu New District, Zunyi, Guizhou China; 3 Respiratory medicine, Sun Yat-Sen Memorial Hospital, Sun Yat-Sen University, Guangzhou, China; 4 Guangdong Provincial Key Laboratory of Malignant Tumor Epigenetics and Gene Regulation, Medical Research Center, Sun Yat-Sen Memorial Hospital, Sun Yat-Sen University, Guangzhou, China; 5 Zhongshan Medical College of Sun Yat-sen University, Guangzhou, China; 6 Breast Tumor Center, Sun Yat-Sen Memorial Hospital, Sun Yat-Sen University, Guangzhou, China

**Keywords:** Cancer metabolism, Metastasis, Breast cancer

## Abstract

MicroRNAs (miRNA) have been shown to be associated with tumor diagnosis, prognosis, and therapeutic response. MiR-328-3p plays a significant role in breast cancer growth; however, its actual function and how it modulates specific biological functions is poorly understood. Here, miR-328-3p was significantly downregulated in breast cancer, especially in patients with metastasis. Mitochondrial carnitine palmitoyl transferase 1a (CPT1A) is a downstream target gene in the miR-328-3p-regulated pathway. Furthermore, the miR-328-3p/CPT1A/fatty acid β-oxidation/stemness axis was shown responsible for breast cancer metastasis. Collectively, this study revealed that miR-328-3p is a potential therapeutic target for the treatment of breast cancer patients with metastasis, and also a model for the miRNA-fatty acid β-oxidation-stemness axis, which may assist inunderstanding the cancer stem cell signaling functions of miRNA.

## Introduction

Breast cancer (BC) is the most common cancer in women [1]. Despite improvements in gene-targeted therapy, radiotherapy, and chemotherapy, the mortality rate of BC remains high [2]. The major cause of cancer-related death in patients with BC is metastasis [3, 4]. Thus, identification of highly metastatic tumors and determining unique therapeutic targets is urgently needed for the treatment of BC.

MicroRNAs (miRNAs) are small, endogenous noncoding RNAs that regulates the expression of downstream genes at the post-transcriptional level by binding the 3’untranslated region (UTR) of the mRNA [5]. MiRNAs have been shown to be associated with tumor diagnosis, prognosis, and therapeutic response, and hold great promise for future cancer diagnosis and treatment [6]. However, due to the heterogeneity among tumors specific miRNAs play different roles in different tumors [7]. Thus, a large area of research is devoted to determining the specific roles of miRNAs in different malignancies.

The expression of miR-328-3p is low in most tumors, such as hepatocellular carcinoma [8], gastric carcinoma [9], and non-small cell lung cancer [10]. Researchers have found that miR-328-3p can downregulate ABCG2 in some BC cell lines, and plays a role in drug resistance [11]. However, other reports have indicated that miR-328-3p can counteract the BC malignant phenotype [12, 13].

In this study, we observed the expression of miR-328-3p was negatively correlated with BC tumor metastasis. Overexpression of miR-328-3p decreased the invasion ability of the highly metastatic MDA-MB-231 and MDA-MB-468 BC cell lines, while inhibiting the expression of miR-328-3p increased the metastasis of BC cells. CPT1A is the target gene of miR-328-3p, which promotes the invasion and metastasis of cancer cells by influencing cellular fatty acid metabolism and regulating cell stemness.

## Materials and methods

### Cell culture and treatment

MCF-10A, MCF-7, MDA-MB-231, and MDA-MB-468 were purchased from American Type Culture Collection (ATCC) and cultured in a cell incubator (37 °C,5% CO_2_) in DMEM medium (GIBCO) supplemented with 10% FBS (GIBCO) and 100 U/ml Penicillin-streptomycin (Life Technologies). Cells were disaggregated using 0.25% Trypsin (Life Technologies) according to the manufacturer’s instructions.

### MiRNA and mRNA target gene analysis

MiR-328-3p expression in breast cancer was analyzed using StarBase V3.0. TargetScan (http://www.targetscan.org/vert_72/), mirWALK (http://mirwalk.umm.uni-heidelberg.de), and mirDB (http://www.mirdb.org) databases.

### Patient samples

Fifty five patient samples were obtained from 2017 to 2019 at the Department of Thyroid and Breast Surgery, Affiliated Hospital of Zunyi Medical University, China. Written consent was approved from the patients enrolled in this study. The pathological information was obtained from the Department of Pathology in Affiliated Hospital of Zunyi Medical University. Samples were all collected within 20 min after resection and then immediately transferred to a −70 °C freezer for use.

### Cell transfection

The mimic NC and mimic miR-328-3p were obtained from Dharmacon (Lafayette, CO, USA), inhibitor NC and inhibitor miR-328-3p were obtained from Life Technologies. Oe-CPT1A, oe-NC and si CPT1A were purchased from GenePharm (Shanghai). The transfection sequences in each group are shown in Table 1. Cells were plated in six-well at 60–70% density and transduced using Lipofectamine 3000 transfection Kit (Invitrogen) following the manufacturer’s instructions.

**Table 1:** The sequences of primers and oligonucleotides used in this study.

Primers	
Hsa-miR-328-3p F	5’-CTGGCCCTCTCTGCCC-3’
Hsa-miR-328-3p R	5’-GTGCAGGGTCCGAGGT-3’
CPT1A F	5’-CTGGACAATACCTCGGAGCC-3’
CPT1A R	5’-AACGTCACAAAGAACGCTGC-3’
GAPDH F	5’-GTCAAGGCTGAGAACGGGAA-3’
GAPDH R	5’-AAATGAGCCCCAGCCTTCTC-3’
U6 F	5′-CUGGCCCUCUCUGCCCUUCCGU-3′
U6 R	5′-CTCGCTTCGGCAGCACATATA-3′

siRNAs targeting sequence	
Si CPT1A 1#	5′-GGATGGGTATGGTCAAGATCT-3′
Si CPT1A 2#	5′-TGCGCCGATCGTGGCCCACCTT-3′

### Dual-luciferase reporter assay

The wild-type and mutant CPT1A 3′-UTR (CCCGGU) were constructed by using the QuickChange II site-directed mutagenesis kit (Stratagene, La Jolla, California, USA) and transfected into the reporter plasmid (Promega, Madison, WI, USA). Cells were co-transfected with plasmids and miR-328-3p mimics or negatively controlled using lipofectamine 3000 (Invitrogen). The firefly and Renilla luciferase activities were measured using the Dual Luciferase Reporter Assay System (Promega, WI, USA) after 48 h. The luciferase activities were normalized to Renilla fluorescence and analyzed by ImageJ.

### Flow cytometry

The cells were disaggregated and resuspended in 1× buffer, with the concentration adjusted to 1 × 10^6^ cells/mL. Fixable Viability Dye eFluor^TM^ 780 (Thermo, Cat#65-0865-14), Anti-CD44 (Biolegend, Cat#338807), Anti-CD24 (Biolegend, Cat#311103) were used protected from light. ALDEFLUOR kit (STEMCELL Technologies) was used according to the manufacturer’s instructions to monitor ALDH1 activity. Samples were detected using CYTEFLEX-2 (Backman).

### Animal experiment

2 × 10^5^ cells treated as Fig. 6 shown were injected into caudal vein of 6-week-old female NOD/SCID mice. For the miR-328-3p intervention, the miR-328-3p agomir or antagonist was intravenously injected through the tail vein for 3 consecutive days (200 nM/Kg/d). After 4 weeks, the mice were anesthetized with an intraperitoneal injection of pentobarbital sodium (40 mg/kg), and surgery was performed. The lung was harvested and given d-luciferin before imaging, and imaged with a Xenogen IVIS Lumina system (Caliper Life Sciences). Subsequently, lung tissues were weighed and fixed in 4% paraformaldehyde and embedded in paraffin for subsequent analysis. For mouse survival analyses, mice were fed for 10 weeks. Metastases were identified via H&E staining and metastatic area was quantified by ImageJ software as a percentage of total lung area.All procedures were approved by the Institutional Review Boards and Animal Care and Use Committees of Zunyi Medical University.

### Immunohistochemistry and immunofluorescence

For immunohistochemistry, tumor tissues were fixed in 4% paraformaldehyde, subsequently embedded in paraffin and routinely cut into 4-μm-thick sections and then mounted on glass slides. Specimens were incubated with antibodies specific for CPT1A (Thermo, PA5-69347, 1:100) overnight at 4 °C and immune detection was performed on the following day using DAB (Dako) according to the manufacturer’s instructions. Staining intensity was quantified by imageJ software. Briefly, the images were converted to 8-bit; the background was subtracted by using the threshold function;

For immunofluorescence, cells were plated on coverslip overnight. The next day, cells were washed two times and stained with BODIPY 493/503 (Thermo, D2184), tumor tissue was performed as previous described and stained with Cytokeratin (1:100, Cat#MNF116, Abcam), and nuclei were stained by DAPI protected from light for 1 h. Then, the cells were washed two times and sealed with Antifade Mounting Medium (Beyotime), and samples were monitored via LSM800 (Zeiss).

For H&E staining, samples were treated as above and stained with a Hematoxylin and Eosin Staining Kit (Beyotime) according to the manufacturer’s instructions. Images were obtained using BX-63 (Zeiss).

### RT-qPCR

Total RNA was extracted from fresh frozen tissues or cells according to the instructions of the Trizol kit (Invitrogen, Carlsbad, CA, USA). The RNA was then dissolved in 1× RNA-enzyme-free diethylpyro carbonate (DEPC). Checking the OD values of RNA at the wavelengths of 260 nm and 280 nm via ND-1000 ultraviolet/visible spectrophotometer (Nanodrop Company, Rockford, IL, USA) to evaluate the quality and concentration of the total RNA. Then cDNA amplification efficiency was evaluated using PrimeScriptTM One Step RT-PCR Kit Ver.2 (TaKaRa) followed the manufacturer’s instructions. Data were analyzed using 2^−ΔCt^ for patient samples and 2^−ΔΔCt^ for cell lines. The primer sequences can be seen in Table 1.

### Transwell assay

The invasion capacities of cells were checked via Transwell assay as described previously [14]. 1 × 10^3^ cells in 200 μL were plated on the upper chambers with 8-μm pore filters (Millipore, Germany) coated with 50 μL Matrigel (BD Biosciences), and 20% FBS was added to the lower chambers as an attractant. upper chamber cells were gently removed and bottom cells were fixed with 4% paraformaldehyde after incubation for 12 h (MDA-MB-231) and 48 h (MCF-7), stained with crystal violet solution for 30 min, and visualized using BX-63 (Zeiss).

### Wound healing assay

Cells were seeded into six-well plates, and 20 μL pipette tip scratched cell monolayer until 70% confluence. The first image was obtained after 0.5 h (avoiding the interference of exfoliated cells). The second image was harvested after 12 h using microscopy (BX-63, Zeiss, Germany). The results are presented as the percentage of wound healing, calculated as follows: [wound area (initial) − wound area (final)/wound area (initial) × 100.

### Western blot

Total protein was extracted from tissues and cells using RIPA buffer and separated by 10% SDS/PAGE gel, then transferred onto PVDF membranes (Millipore, Billerica, MA). Membranes were blocked with nonfat milk for 1 h and stained with Anti-CPT1A (Thermo, Cat# PA5-69347, 1:1000), Anti-ALDH1 (CST, Cat#36671, 1:1000), and HRP-GAPDH (Abcam, ab9484, 1:3000) overnight at 4 °C. The next day, the membranes were washed three times and stained with IgG H&L (HRP) (Abcam, ab7090, 1:3000) for 2 h. The antigen–antibody reactions were visualized with enhanced chemiluminescence assays (ECL, Thermo). The images were cropped for presentation via photoshop.

### Seahorse metabolic flux analysis

Oxygen consumption rate (OCR) was measured using the XF Cell Mito Stress test (Santa Clara, CA, USA) as previous reported [15]. Briefly, Oligomycin (selleck.cn, S1478), FCCP (MCE, HY-100410), and the compound of rotenone (Selleck.cn, S2348) and antimycin A (Santa Cruz Biotechnology, CAS 1397-94-0) to modulate the electron transport chain. 4 × 10^4^ cells were treated overnight with no serum. The next day, 50 μM palmitate-BSA were supplemented 2 h before the assay. The assay media must be no glucose and bicarbonate, pH 7.4. Analysis was performed with Seahorse Wave software (Agilent, Santa Clara, CA, USA).

### Mammosphere formation and treatment

Mammosphere formation was conducted in MCF-7 and MDA-MB-468 cells via stem cell media (DMEM/F12 (Gibco), 2% B27 (Life Technologies), 20 ng/mL hEGF (PeproTech), 20 ng/mL hFGF2 (PeproTech)). One thousand cells were cultured in ultralow adhesion plates (Corning), and mammospheres were counted by ImageJ software after 10 days. In some experiments, cells were treated with inhibitor or mimic mir-328-3p before mammosphere formation assay.

### Cell proliferation assay

Cell proliferation assay was performed using colony-formation assay or a CCK8 kit (Dojindo Laboratories, Kumamoto, Japan) following the manufacturer’s instructions. Ten thousands cells in 100 mL were incubated in 96-well plates for 48 h, cells in each well were treated with the CCK8 reagent and incubated at 37 °C for 2 h. The optical density (OD) at 450 nm was read using a microplate reader (Tecan, Switzerland). For colony-formation assay, 500 cells were seeded into six-well plates and then incubated for 14 days. Then, the colonies were fixed with 4% paraformaldehyde for 15 min and stained with 0.1% crystal violet (Cat#HT90132, Sigma–Aldrich) for 15 min at room temperature. The cell colonies were counted and photographed.

### Statistical analysis

Statistical analysis was processed by using the Graphpad Prism 7 software (RRID:SCR 000306). Student’s *t*-test (two-tailed) and one-way ANOVA were used to detect differences between two groups and more than two groups. Results of western blot and wound healing assays were quantified using ImageJ software (National Institutes of Health). The overall survival rates of mice in each group were estimated suing the Kaplan–Meier method. All data are shown as the mean ± standard deviation (SD). A value of *p* < 0.05 was considered to be indicative of statistical significance.

## Results

### MiR-328-3p expression is negatively correlated with BC metastasis

**Fig. 1:**
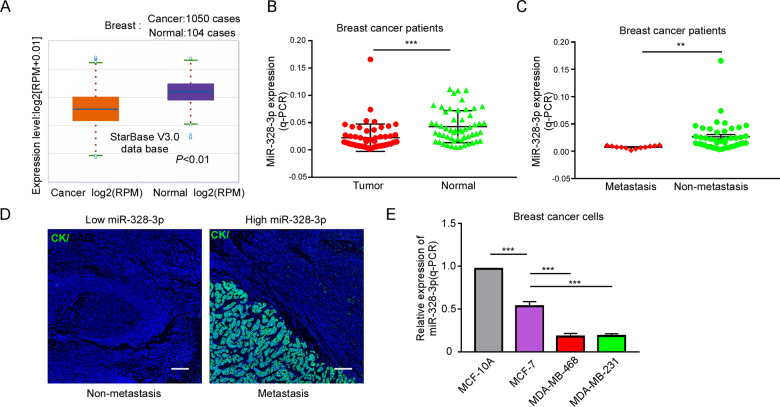
MiR-328-3p was expressed in BC and inverse association with metastasis. **A** StarBase V3.0 database analyzes miR-328-3p expression in normal breast and BC tissues. **B** The expression of miR-328-3p in clinical BC and adjacent normal tissues of 55 BC patients by RT-qPCR, ***p < 0.001 by Student’s *t*-test. **C** The expression of miR-328-3p levels in nonmetastatic and metastatic status of BC patients. Data are shown as mean ± SD, **p < 0.01 by Student’s *t*-test. **D** The representative images of cytokeratin (CK) expression in lymph node of low and high miR-328-3p expression BC. Scale bar: 100 μm. **E** The relative expression of miR-328-3p in the indicated BC cell lines. Data are shown as mean ± SD, ***p < 0.001 by student’s *t*-test (*n* = 3). The results were normalized to MCF-10A cells.

To validate the role of miR-328-3p in BC, we first used StarBase v3.0 to analyze miR-328-3p expression and found that it was significantly decreased in BC tissues compared to that in normal tissues (Fig. 1A). Then, tissue specimens from 55 BC patients who underwent surgical resection at the first affiliated hospital of Zunyi Medical University were examined. The expression of miR-328-3p was examined using RT-qPCR in BC tissues and adjacent normal tissues, and the results showed that the expression of miR-328-3p was decreased 72.7% (40/55) in cancer tissues compared to adjacent normal tissues (Fig. 1B). In addition, 12 patients with metastasis (lung and/or liver) had lower miR-328-3p expression than the 43 patients without metastasis (Fig. 1C), indicating that miR-328-3p expression is negatively correlated with BC metastasis. Patient characteristics are summarized in Table 2. Interestingly, this phenomenon was only observed with distant metastasis, but not lymphatic metastasis (Fig. 1D).

**Table 2:** Characteristics of the studied BC patients.

Characteristics	*N*
Age	
>50 years	19
≤50 years	36
Molecular subtyping	
LuminaA	6
LuminaB	25
Triple negative	8
HER2+	16
Lymph node metastasis	
Yes	35
No	20
Distant metastasis	
Negative	43
Positive	12
TNM staging system	
T1 + T2	40
T3 + T4	15

Consistently, normal breast epithelial cells (MCF-10A) contain higher miR-328-3p expression compared with BC cell lines (MCF-7,MDA-MB-231 and MDA-MB-468). Similarly, miR-328-3p was downregulated in BC cells with high metastatic potential (MDA-MB-231 and MDA-MB-468) compared to those with low-metastatic potential (MCF-7) (Fig. 1E). Together, these results suggest that miR-328-3p negatively regulates BC progression, especially metastasis.

### MiR-328-3p attenuates the invasion and migration of BC

**Fig. 2:**
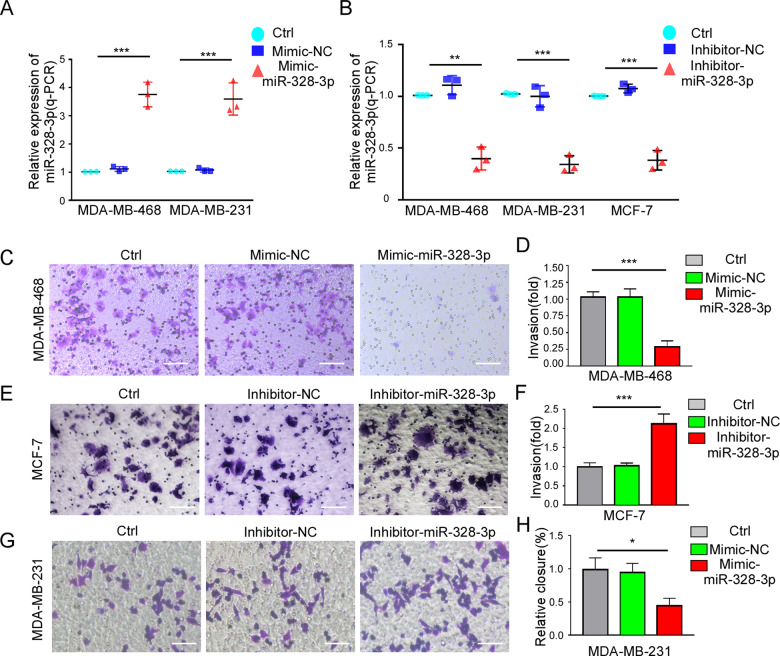
MiR-328-3p overexpression dampens breast cancer metastasis. **A** The relative expression of miR-328-3p in MDA-MB-468 and MDA-MB-231 cells with mimic-miR-328-3p. Mean ± SD, ***p < 0.001 by one-way ANOVA with Dunnett *t*-test, *n* = 3. **B** The relative expression of miR-328-3p in MDA-MB-468, MDA-MB-231 and MCF-7 with inhibitor miR-328-3p. Mean ± SD, **p < 0.01, ***p < 0.001 by one-way ANOVA with Dunnett *t*-test, *n* = 3. **C, D** The invasion ability of MDA-MB-468 cells transfected with mimic-miR-328-3p was detected by Transwell assay. Panel **C** is a representative images, and panel **D** is a relative statistic graph. Scale bar: 50 μm. Mean ± SD, ***p < 0.001 by one-way ANOVA with Dunnett *t*-test, *n* = 3. **E, F** The invasion ability of MCF-7 with miR-328-3p inhibition was detected by the Transwell assay. Representative images (**E**) and statistical graphs (**F**) are shown. Bar: 50 μm. Mean ± SD, ***p < 0.001 by one-way ANOVA with Dunnett *t*-test, *n* = 3. **G** The invasion ability of MDA-MB-231 with miR-328-3p inhibition was detected by the Transwell assay. Bar: 50 μm. **H** Wound closure was evaluated using the equation described in the materials and methods. Values are the mean ± SD of three independent experiments. *p < 0.05 by one-way ANOVA with Dunnett *t*-test.

To clarify the relation between miR-328-3p and BC metastasis, BC cells were transfected with mimic miR-328-3p to increase the expression of miR-328-3p, or transduced with inhibitor miR-328-3p to interrupt miR-328-3p expression (Fig. 2A, B). Transwell assays and scratch wound healing assays were performed to detect whether a change in miR-328-3p expression affects the invasion ability of BC cells. We found that upregulation of miR-328-3p significantly impaired the invasion capacity of BC cells as compared to that of the control group in Transwell assay (Fig. 2C, D, Supplementary Data 1A, B). In contrast, downregulation of miR-328-3p increased the invasive ability of BC cells (Fig. 2E–G, Supplementary Data 1C). The results of the scratch wound healing assay were similar (Fig. 2H, Supplementary Data 1D).

The CCK8 assay and colony-formation assay were performed to examine the relation between miR-328-3p and cell proliferation. The results showed that miR-328-3p interrupted cancer cell proliferation in 10% fetal bovine serum (FBS) medium, but not in stem cell medium (Supplementary Data 1E, F), which is consistent with prior reports [16, 17]. Taken together, the results indicate that miR-328-3p attenuates the invasive ability of BC cells.

### CPT1A is a downstream target of miR-328-3p in BC

**Fig. 3:**
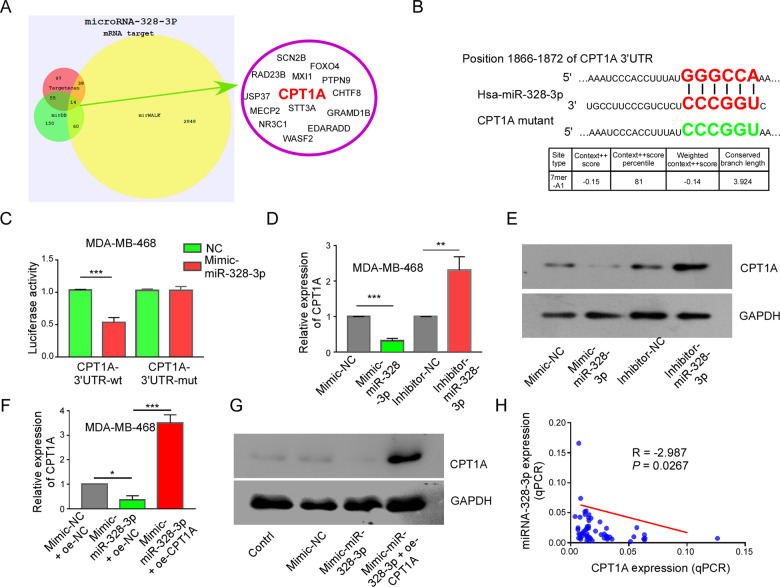
CPT1A serves as a downstream target of miR-328-3p in BC. **A** The target genes of miR-328-3p were analyzed by TargetScan, mirWALK, and mirDB database. Violet circle showed intersection gene of three database. **B** The binding position of miR-328-3p to CPT1A was analyzed by TargetScan website, and mutant sites of CPT1A was shown. **C** The effect of mimic-miR-328-3p on the CPT1A-wt and CPT1A-mut reporters in MDA-MB-468 cell lines was measured by dual-luciferase reporter gene. Mean ± SD, ***p < 0.001 by student’s *t-*test, *n* = 3. **D, E** The relative expression of CPT1A in MDA-MB-468 cells with miR-328-3p overexpression or knockdown was detected via RT-qPCR (**D**) and western blot (**E**). Mean ± SD, **p < 0.01,***p < 0.001 by student’s *t*-test (*n* = 3). **F** The relative expression of CPT1A in MDA-MB-468 cells transfected with mimic-miR-328-3p and/or CPT1A overexpression (oe-CPT1A). Mean ± SD, *p < 0.05, ***p < 0.001 by one-way ANOVA with Dunnett *t*-test, *n* = 3. **G** MCF-7 cells were treated as **F**, the CPT1A expression was detected by western blot, *n* = 3. **H** The relation of miRNA-328-3p and CPT1A in 55 breast cancer patients was shown.

MiRNAs can interact with mRNAs to regulate cell function. Therefore, we analyzed the target genes of miR-328-3p via the TargetScan, mirWALK, and mirDB databases and found 14 genes that intersect in the three databases, as shown in Fig. 3A. We chose CPT1A as the target gene for this study for the following reasons: (1) The databases predict that CPT1A has a high interaction score with miR-328-3p. (2) CPT1A has been reported to influence the malignant behavior of BC, although the mechanism is poorly understood [18]. (3) The regulatory effect of miR-328-3p on CPT1A is unknown.

CPT1A is a mitochondrial protein that transfers long-chain fatty acids into the mitochondria for oxidation [19]. The database search indicated that position 1866–1872 of CPT1A 3’UTR links with CCCGGU of miR-328-3p (Fig. 3B). To study the direct relation between miR-328-3p and CPT1A, we constructed dual-Luciferase reporters containing the 3’UTR of CPT1A and the mutant-type 3’UTR of CPT1A (Fig. 3B). The results shown that mimic miR-328-3p significantly decreased the Luciferase activity of CPT1A wild-type reporters, but not the mutant-type (Fig. 3C). In addition, the change in CPT1A protein was detected by immunoblotting in MDA-MB-468 cells with silenced and overexpressed miR-328-3p. The results showed that the expression of CPT1A was significantly increased when miR-328-3p was knocked down, but it was decreased when miR-328-3p was overexpressed (Fig. 3D, E). Further experiments indicated that CPT1A upregulation neutralized the function of mimic miR-328-3p that reduced the expression of CPT1A (Fig. 3F, G). The results illustrated that miR-328-3p inhibits the expression of CPT1A via promoting mRNA decay, not by inhibiting protein translation. Consistently, similar results were found in experiments using MCF-7 and MDA-MB-231 cells (Supplementary Data 2A–D). In addition, miR-328-3p expression was negatively correlated with CPT1A in the 55 BC patients (Fig. 3H). The above results demonstrate that miR-328-3p directly binds and negatively regulates the expression of CPT1A by decreasing CPT1A mRNA.

### CPT1A facilitates BC metastasis by regulating miR-328-3p

**Fig. 4:**
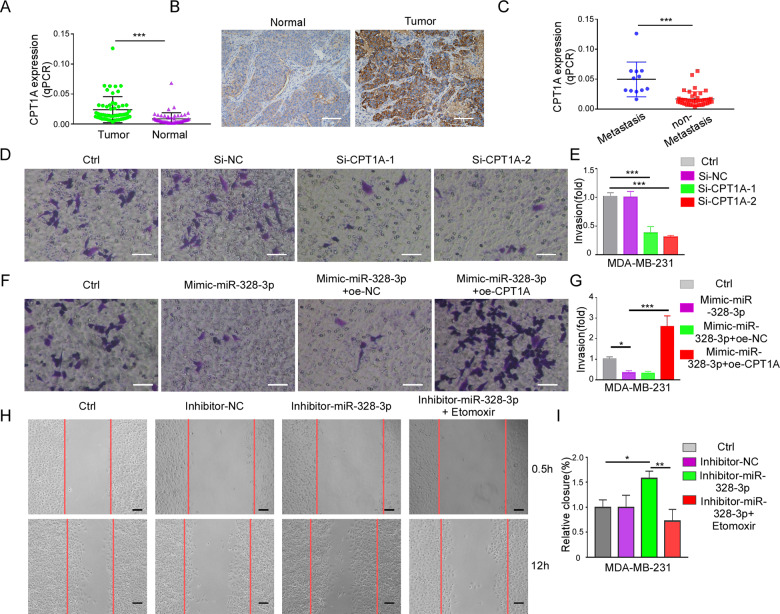
CPT1A facilitates BC cells invasion and migration was regulated by miR-328-3p. **A** The expression of CPT1A in BC tissue and adjacent normal tissue by RT-qPCR. Data are presented as mean ± SD, ***p < 0.001 by student’s *t*-test. **B** Representative image of immunohistochemical staining with CPT1A antibody in BC and adjacent normal tissues. Scale bar: 20 μm. **C** The expression of CPT1A in metastatic and nonmetastatic BC patients. Mean ± SD, ***p < 0.001 by student’s *t*-test. **D, E** The invasion ability of MDA-MB-231 with CPT1A downregulation was detected by Transwell assay. Representative images (**D**) and relative statistical graphs (**E**) are shown. Scale bar: 50 μm. Mean ± SD,***p < 0.001 by one-way ANOVA with Dunnett *t*-test (*n* = 3). **F, G** The invasion ability of MDA-MB-231 transfected with mimic-miR-328-3p with or without CPT1A overexpression was detected by Transwell assay. Representative images (**F**) and relative statistic graphs (**G**) are shown. Scale bar: 50 μm. Mean ± SD, *p < 0.05, ***p < 0.001 by one-way ANOVA with Dunnett *t*-test (*n* = 3). **H, I** Scratch wound healing assay in MDA-MB-231 cells transduced with miR-328-3p inhibitor with or without Etomoxir. Representative images (**H**) and relative statistic graphs (**I**) are shown. Scale bar: 100 μm. Mean ± SD, *p < 0.05, **p < 0.01 by one-way ANOVA with Dunnett *t*-test (*n* = 3).

CPT1A is highly expressed in multiple tumors [20–22], but whether CPT1A plays function in BC metastasis is not clear. To determine the role of CPT1A in BC metastasis, we used RT-qPCR and immunohistochemistry to examine the expression of CPT1A in BC patients and BC cell lines. The results showed that the expression of CPT1A was higher in cancer tissue than adjacent normal tissue (Fig. 4A, B, Supplementary Data 3A, B), and was higher in BC patients with metastasis than those without metastasis (Fig. 4C). Kaplan–Meier analysis showed that high expression of CPT1A is associated with distant metastasis-free survival and overall survival of patients with BC (Supplementary Data 3C, D).

To study the direct relation between CPT1A and BC metastasis, we downregulated CPT1A expression by silencing CPT1A mRNA (Supplementary Data 3E) and found that CPT1A knockdown significantly impaired the invasion capacity of BC cells as compared to the control group in Transwell assay (Fig. 4D, E). Furthermore, the effect of mimic miR-328-3p on the metastatic capability of BC cells could be rescued by CPT1A overexpression (Fig. 4F, G; Supplementary Data 3F). Consistent with the above results, the scratch wound healing assay showed that metastatic ability of BC cells treated with miR-328-3p inhibitor can be rescued by treatment with etomoxir, a CPT1A inhibitor [23] (Fig. 4H, I). Collectively, the above results indicate that miR-328-3p attenuates BC metastasis by regulating CPT1A.

### MiR-328-3p controls fatty acid β-oxidation via CPT1A

**Fig. 5:**
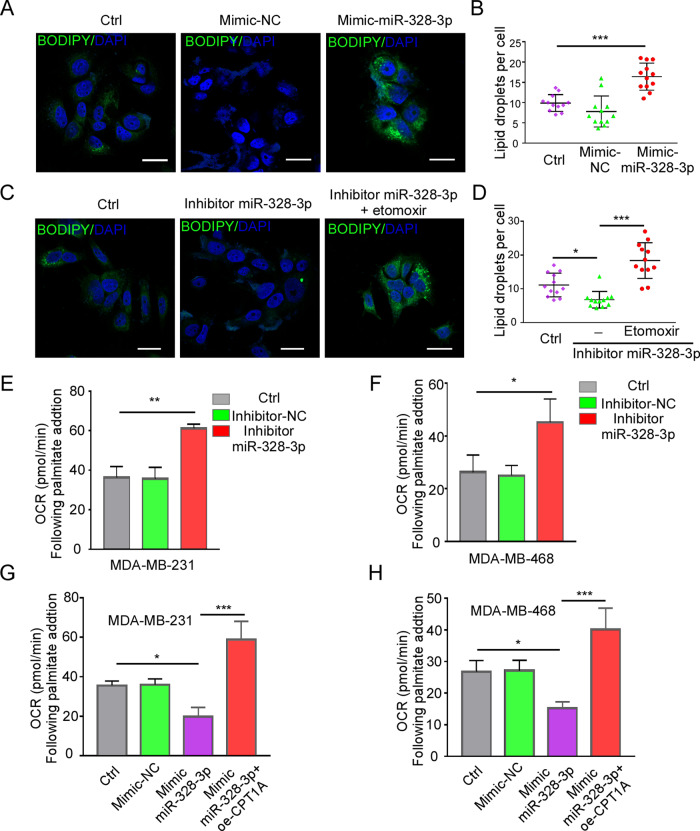
MiR-328-3p controls fatty acid β-oxidation via CPT1A. **A, B** Lipid droplet levels in MDA-MB-468 cells with mimic-miR-328-3p was detected by BODIPY 493/503 staining. Representative images are shown in **A**, the fluorescence foci were quantified and shown in **B**. Scale bar: 20 μm. Mean ± SD, ***p < 0.001 by one-way ANOVA with Dunnett *t*-test, *n* = 12 in three independent experiment. **C, D** The lipid droplet levels in MDA-MB-468 cells transduced with inhibitor miR-328-3p with or without Etomoxir were evaluated by BODIPY 493/503 staining. Representative images (**C**) and fluorescence foci quantification (**D**) are shown. Scale bar: 20 μm. Mean ± SD, *p < 0.05, ***p < 0.001 by one-way ANOVA with Dunnett *t*-test, *n* = 12 in three independent experiment. **E–H** Change in OCR (ΔOCR) following palmitate-BSA addition calculated as (OCR at the time of palmitate-BSA injection—final basal OCR) in MDA-MB-231 and MDA-MB-468 with miR-328-3p knockdown (**E**, **F**) or overexpression (**G, H**). Mean ± SD, *p < 0.05, **p < 0.01, ***p < 0.001 by one-way ANOVA with Dunnett *t*-test (*n* = 3).

Fatty acid β-oxidation (FAO) is critical for survival and proliferation of cancer cells [24–26]. In the FAO process, the shuttle of long-chain fatty acyl-CoA (LCA CoA), which becomes acetyl-CoA and undergoes several rounds of β-oxidation to generate ATP in the tricarboxylic acid (TCA) cycle [19]. Acetyl-CoA enters mitochondria via CPT1 is the first and rate-limiting step, the CPT1A is a crucial member of CPT1 family. Based on these data, we hypothesized that miR-328-3p regulates the rate of mitochondrial FAO, and this could be the pathway by which miR-328-3p is involved in BC metastasis. To test this idea, we used BODIPY 493/503 staining to monitor changes in intracellular lipid levels of BC cells with miR-328-3p overexpression and found that miR-328-3p overexpression significantly enhanced intracellular lipid droplet levels, but this was reversed by CPT1A overexpression (Fig. 5A, B, Supplementary Data 4A, B). In contrast, treatment with miR-328-3p inhibitor reduced intracellular lipid droplet levels, but this was reversed by CPT1A antagonists (Fig. 5C, D).

To further verify that miR-328-3p regulates fatty acid metabolism, seahorse metabolic analyzer was used to measure mitochondrial bioenergetics of BC cells with miR-328-3p alterations when given palmitate-BSA. We observed that the basal respiration rate was significantly elevated in miR-328-3p knockdown cells, but decreased in miR-328-3p upregulated cells after addition of fatty acid (Fig. 5E-H, Supplementary Data 4C, D). These findings suggest that miR-328-3p interrupts BC cell fatty acid metabolism via regulation of CPT1A.

### Overexpression of miR-328-3p suppresses cancer metastasis by interrupting FAO in vivo

**Fig. 6:**
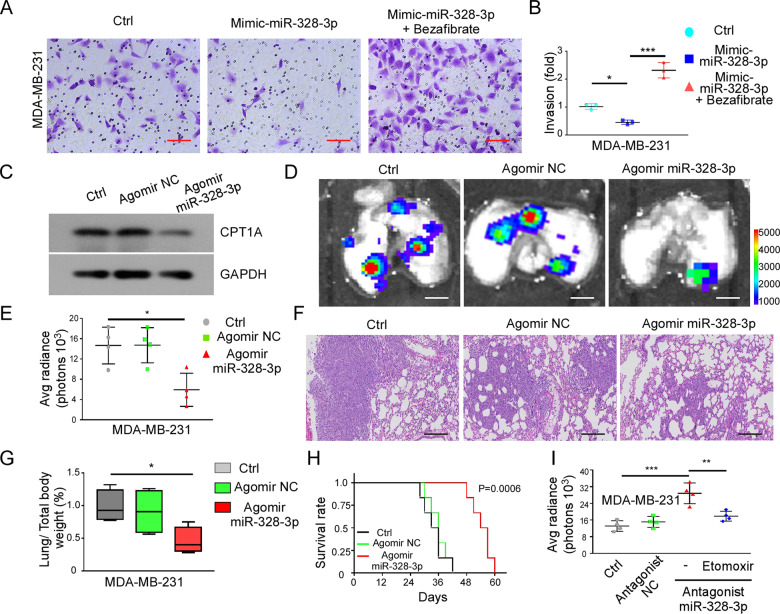
Overexpression of miR-328-3p represses cancer metastasis by interrupting fatty acid β-oxidation in vivo. **A, B** The relative invasion ability of MDA-MB-231 cells transfected with mimic-miR-328-3p with or without Bezafibrate, a FAO agonist, was evaluated by Transwell assay. Representative images (**A**) and relative statistic graph (**B**) are shown. Scale bar: 50 μm. Mean ± SD, *p < 0.05, ***p < 0.001 by one-way ANOVA with Dunnett *t*-test (*n* = 3). **C** The expression of CPT1A in MDA-MB-231 cells treated with miR-328-3p agomir was analyzed by immunoblotting. **D** Nod/scid mice were injected intravenously with 2 × 10^5^ MDA-MB-231 cells treated with miR-328-3p agomir (*n* = 4/group). Lung tumor burden was assessed at the end of the experiment (day 28) by ex vivo IVIS imaging. Scale bar: 50 μm. **E** The average radiance of lung metastasis foci in **D**. Mean ± SD, *p < 0.05 by one-way ANOVA with Dunnett *t*-test (*n* = 4/group). **F** Metastasis tumor area from three sections cut through the lungs 150 μm were detected by H&E staining. Scale bar: 10 μm. **G** The ratio of lung weights to total body weight of Nod/scid mice. Mean ± SD, *p < 0.05 by one-way ANOVA with Dunnett *t*-test (*n* = 4/group). **H** The overall survival rates of mice in each group were estimated by the Kaplan–Meier method (*n* = 6/group). **I** The average radiance of lung metastasis foci in MDA-MB-231 with or without antagonist miR-328-3p and Etomoxir. Mean ± SD, **p < 0.01, ***p < 0.001 by one-way ANOVA with Dunnett *t*-test (*n* = 4/group).

FAO is one of the main sources of ATP production, but recent studies have shown that FAO is involved in the malignant behavior of tumors, and is one of the mechanisms of tumor progression [24]. Previous results indicated that overexpression of miR-328-3p suppresses the metastasis of liver cancer by regulating CPT1A [17]. Whether miR-328-3p is related to the FAO pathway in BC metastasis is unknown. To answer this question, we added bezafibrate, a FAO agonist [24], to cells treated with mimic miR-328-3p and found that bezafibrate interrupted the function of mimic-miR-328-3p (Fig. 6A, B). To test if similar results would be found in vivo, we constructed a miR-328-3p agomir, which increased miR-328-3p expression (Fig. 6C) and found that the miR-328-3p agomir resulted in fewer lung metastasis of BC cells than the control treatment (Fig. 6D, E). In addition, examination of lung metastatic lesions by H&E staining and lung weight provided similar results (Fig. 6F, G, Supplementary Data 5). Moreover, miR-328-3p agomir significantly improved survival time as compared to that of the control (Fig. 6H). In contrast, treatment with a miR-328-3p antagonist increased the number of lung metastasis of BC cells, but the effect was reversed by inhibition of FAO (Fig. 6I). Together, these results indicate that miR-328-3p decreases BC metastasis by regulating the FAO pathway.

### MiR-328-3p depletion enhances BC cell stemness

**Fig. 7:**
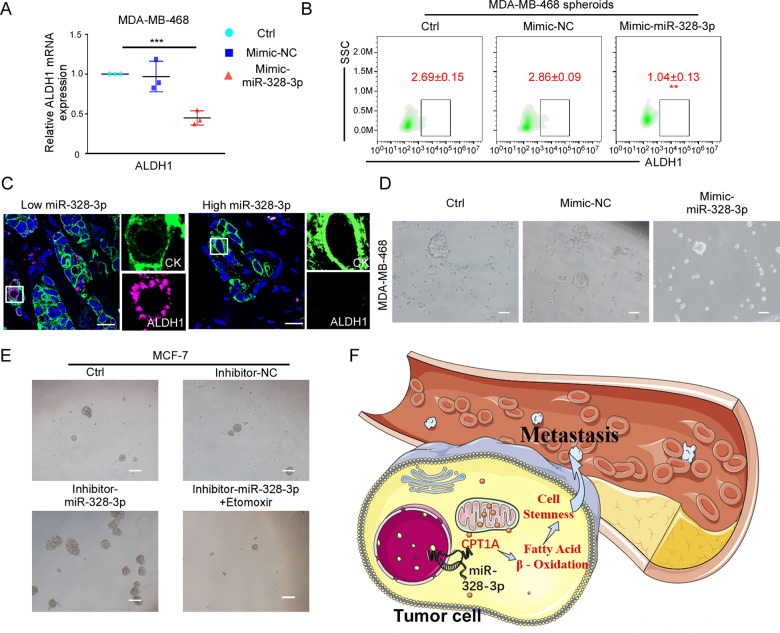
MiR-328-3p depletion enhances cancer cells stemness. **A** The relative ALDH1 expression in MDA-MB-468 cells with overexpressed miR-328-3p by RT-qPCR. Mean ± SD, ***p < 0.001 by one-way ANOVA with Dunnett *t*-test (*n* = 3). **B** The proportions of ALDH1+ cancer cells were monitored by flow cytometry, cells were treated with or without mimic-miR-328-3p in MDA-MB-468 CSCs spheroids. Mean ± SD, **p < 0.01 by One-way ANOVA with Dunnett *t*-test (*n* = 3). **C** The co-expression of cytokeratin (CK) and ALDH1 in clinical BC tissue with low or high miR-328-3p expression. Scale bar: 50 μm. **D, E** The spheroid forming ability of MDA-MB-468 (**D**) or MCF-7 (**E**) in the indicated treatment. Scale bar: 30 μm. **F** Scheme of the miR-328-3p-mediated cell stemness network in BC: The low expression of miR-328-3p in small partition BC patients who suffered tumor metastasis, which promotes CPT1A expression that enhances fatty acid β-oxidation and stemness of cells, then promotes cancer cell metastasis.

Tumor metastasis involves various factors, and cancer stem-like cells (CSCs) are one of the main mechanisms of tumorigenesis and cancer progression [27, 28]. It has been reported that FAO participates in the regulation of cancer cell stemness [24]. Therefore, we speculated that miR-328-3p inhibits tumor metastasis via the FAO-stemness pathway. To test this hypothesis, we first detected CSCs marker expression and activity, and found that miR-328-3p overexpression decreased the stem cell marker ALDH1 mRNA and protein level compared to control (Fig. 7A, B). Moreover, miR-328-3p was negatively correlated with ALDH1 in BC patients (Fig. 7C, Supplementary Data 6A). The regulation is not direct, because the change of FAO can interrupt the ALDH1 regulation by miR-328-3p (Supplementary Data 6B). In further experiments, we found that inhibition of miR-328-3p dramatically increased the proportion of CD44 + CD24-cells (cancer stem-like cells). Surprisingly, FAO metabolism inhibitor interrupted the CD24 regulation of miR-328-3p, but not CD44 regulation (Supplementary Data 6C-E). The above results indicate that cancer stemness was regulated by miR-328-3p is an indirect process.

To further verify the relation between miR-328-3p and cancer stemness, we increased miR-328-3p expression and found that upregulated miR-328-3p expression lessened the 3D spherical-forming ability of cells (Fig. 7D, Supplementary Data 6F). Consistently, downregulation of miR-328-3p promoted the 3D spherical-forming ability, and the effect was reversed by treatment with etomoxir (Fig. 7E, Supplementary Data 6G). These results indicate that miR-328-3p dampens the stemness characteristics of tumors by regulating the FAO pathway.

## Discussion

Metastasis is the main cause of death in BC patients, and thus finding and inhibiting metastasis regulators and pathways can reduce the mortality of BC patients. MiRNAs have been defined as therapy targets in various cancers [7, 29, 30]. Although miR-328-3p has been reported to be important in the regulation of BC cells, studies have provided contradictory results and specific mechanisms have not been identified [11–13]. In this study, we found that the expression of miR-328-3p is lower in BC tissue than adjacent normal tissue. Interestingly, miR-328-3p is highly expressed in BC patients without metastasis as compared to low expression in patients with metastasis, which indicates that miR-328-3p is involved in BC metastasis. Subsequently, we discovered that miR-328-3p agomir reduced the number of lung metastatic foci of BC cells and lung weight in a model of lung metastasis. Previous studies have reported that miR-328-3p inhibits the proliferation of cancer cells with 10% FBS medium, which was coincide with our results, but we can not found that similar phenomenon in cancer cells with stem cell medium. The results suggested that miR-328-3p own different function in various conditions. However, how miR-328-3p is regulated to modulate specific biological functions is poorly understood.

Next, based on information from three databases, CPT1A was studied as a target gene of miR-328-3p. CPT1A is a member of the CPT1 family, and the key enzyme required for carnitine-dependent transport across the mitochondrial inner membrane, and deficiency results in a decreased rate of FAO [19]. Recently, the function of CPT1A has drawn increasing attention with respect to cancer progression including metastasis [31] and radiation-resistance. Our results showed that CPT1A regulation by miR-328-3p enhances BC metastasis in vivo and in vitro.

As previously described, CPT1A is a regulator of FAO and increases both cancer growth and metastasis. Therefore, we sought to explore if miR-328-3p inhibits cancer metastasis by controlling the FAO pathway in cancer cells. Interestingly, mimic miR-328-3p enhanced lipid droplet deposition and dampened the FAO pathway as shown by BODIPY staining and OCR measurement. The overall results indicated that miR-328-3p suppresses the FAO process by regulating CPT1A.

Prior study has shown that FAO promotes BC cell stemness via CPT1B [24]. However, whether the FAO pathway is regulated by miR-328-3p-CPT1A in a similar manner is unknown. Here, we found that inhibitor miR-328-3p apparently enhanced the stemness of BC cells by regulating FAO pathway. The stemness marker were indirectly regulated by miR-328-3p, because the change of FAO metabolism interrupted the regulation of miR-328-3p for CSCs marker, except for CD44. In addition, the 3D spherical-forming ability experiments showed miR-328-3p can regulate BC cell stemness via the CPT1A-FAO pathway. Together, these results confirm that miR-328-3p inhibits BC cell malignant characteristics, which is consistent with study that has shown miR-328-3p is involved in CSCs stemness regulation in colorectal cancer [32]. Recently, a study reported that inhibition of miR-328-3p impairs ovarian CSCs function, a finding that is inconsistent with our results [33]. For this reason, we examined relevant literature and found that miR-328-3p has different roles in different human cancers [8–10, 12, 32, 33], indicating that the function of miR-328-3p could be tissue and tumor specific.

In summary, our results demonstrate that the miR-328-3p-CPT1A-FAO axis plays a critical role in BC metastasis by regulating BC cell stemness. Thus, upregulated miR-328-3p or interruption of the axis can be exploited for the development of efficient strategies for decreasing metastasis in BC patients.

## Supplementary information

Supplementary figures and legends

primary WB
