# Supplementary material for: Fatty acid β-oxidation promotes breast cancer stemness and metastasis via the miRNA-328-3p-CPT1A pathway
Source: Cancer Gene Ther. 2021 May 27;29(3-4):383–95. doi: 10.1038/s41417-021-00348-y (PMC8940624; doi:10.1038/s41417-021-00348-y)
Supplement: Supplementary file 1 — Supplementary figures and legends [file 41417_2021_348_MOESM1_ESM.docx]

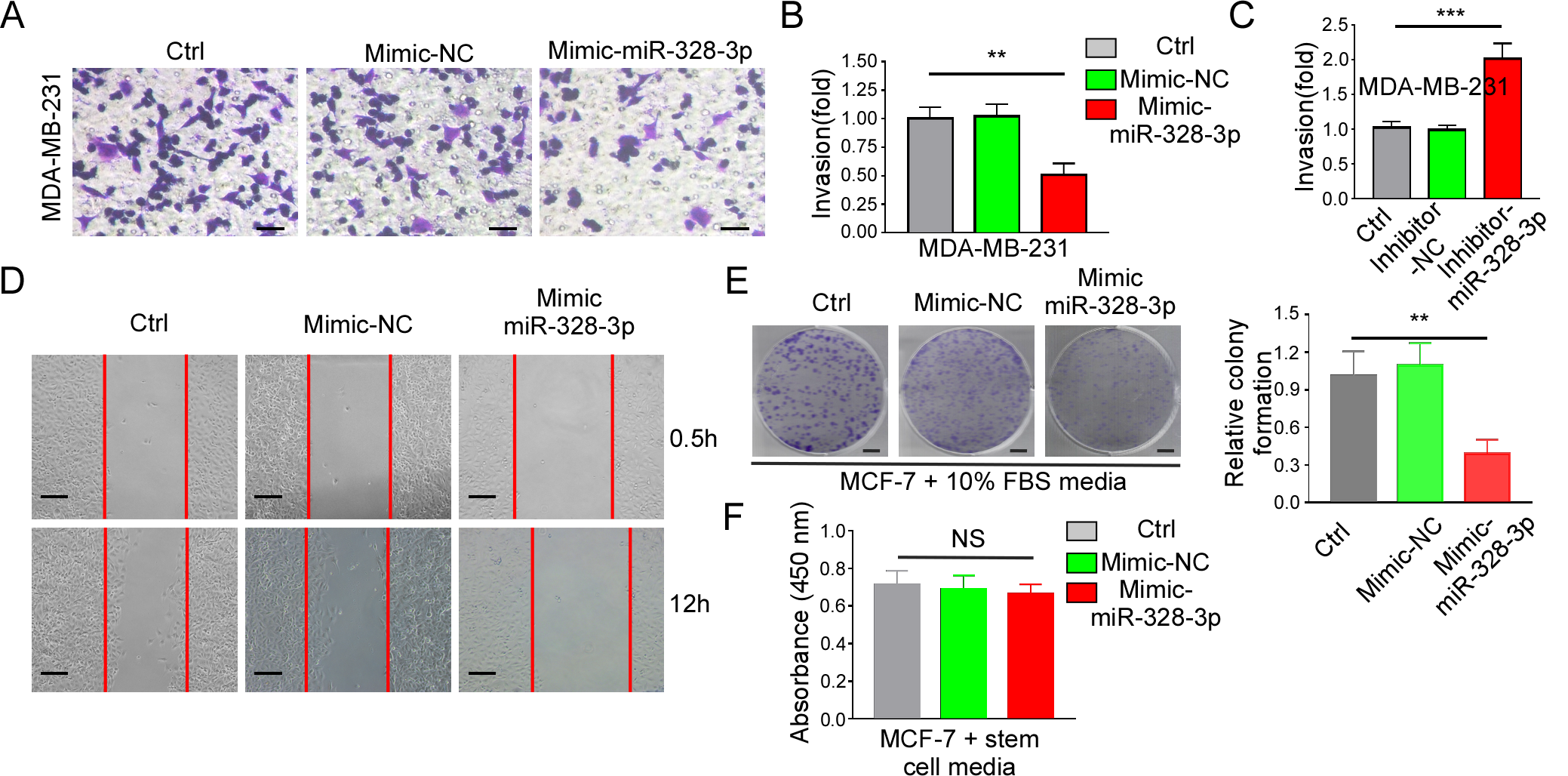


**Supplementary Data 1: MiR-328-3p interrupts breast cancer metastasis *in vitro***

**A and B,** The invasion ability of MDA-MB-231 cells transfected with mimic-miR-328-3p were detected by Transwell assay. (A) is representative image, (B) is relative statistic graph. Scale bar: 50 μm. Mean ± SD, ***p* < 0.01 by One-Way ANOVA with Dunnett-t test, n = 3.

**C,** The statistic in Fig. 2G. ****p* < 0.001 by One-Way ANOVA with Dunnett-t test, n = 3.

**D,** Scratch wound healing assay in MDA-MB-231 cells with miR-328-3p overexpression. Scale bar: 100 μm.

**E,** The typical images of colony formation assay (left) and relative colony formation rate (right) were shown, ***p* < 0.01 by One-Way ANOVA with Dunnett-t test, n = 3 .

**F,** Proliferation ability of MCF-7 with miR-328-3p overexpression, which were cultured in stem cell medium, was measured by CCK8. NS, no significant, n = 3 .


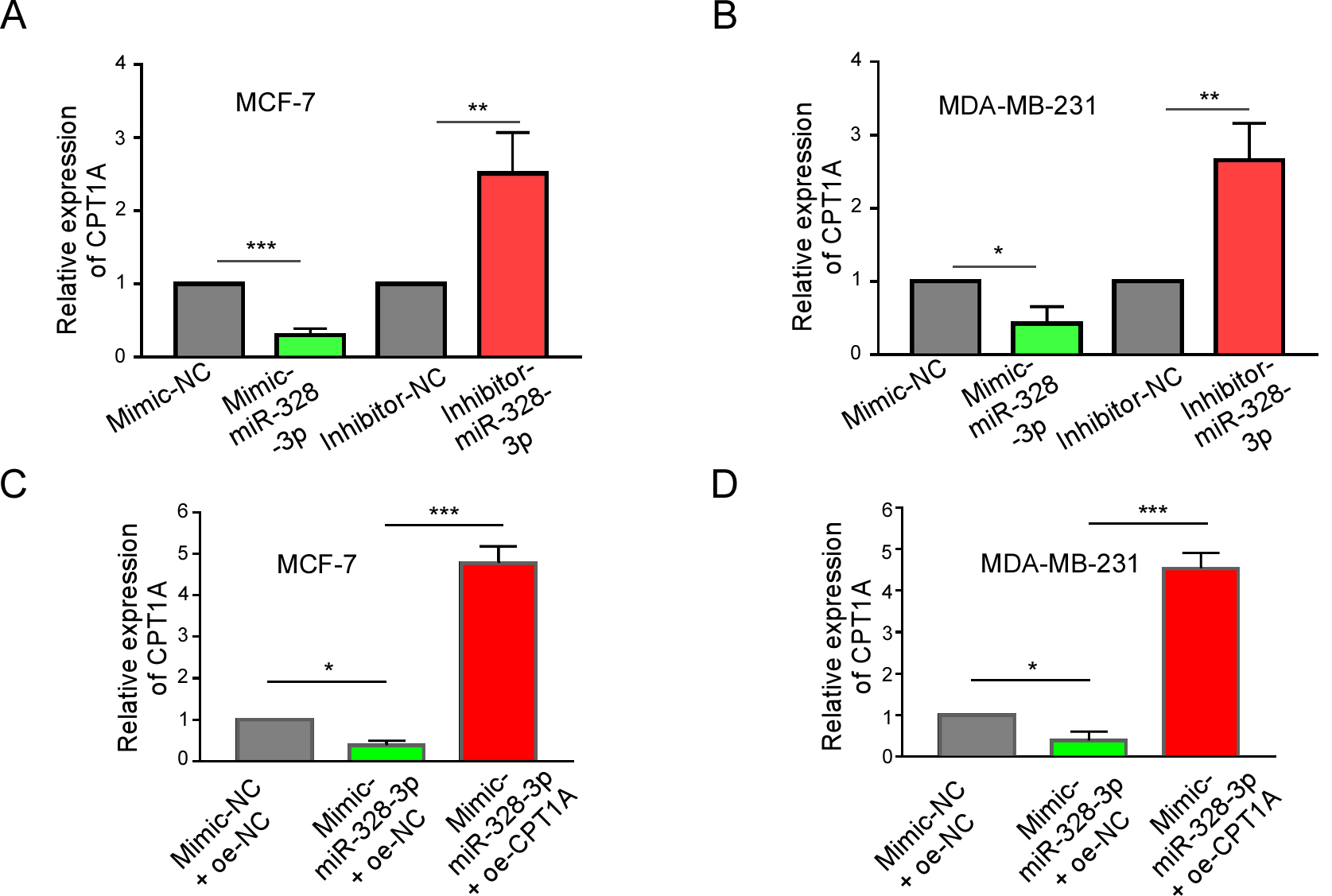


**Supplementary Data 2: CPT1A is a down stream protein of miR-328-3p**

**A and B,** The relative expression of CPT1A in MCF-7 (A) and MDA-MB-231 (B) cells with miR-328-3p overexpression or knockdown was detected via RT-qPCR. Mean ± SD, **p* < 0.05, ***p* < 0.01, ****p* < 0.001 by student’s t test (n = 3).

**C and D,** The relative expression of CPT1A in MCF-7 (C) and MDA-MB-231 (D) cells transfected with mimic-miR-328-3p with or without CPT1A overexpression was detected via RT-qPCR. Mean ± SD, **p* < 0.05, ****p* < 0.001 by One-Way ANOVA with Dunnett-t test (n = 3).


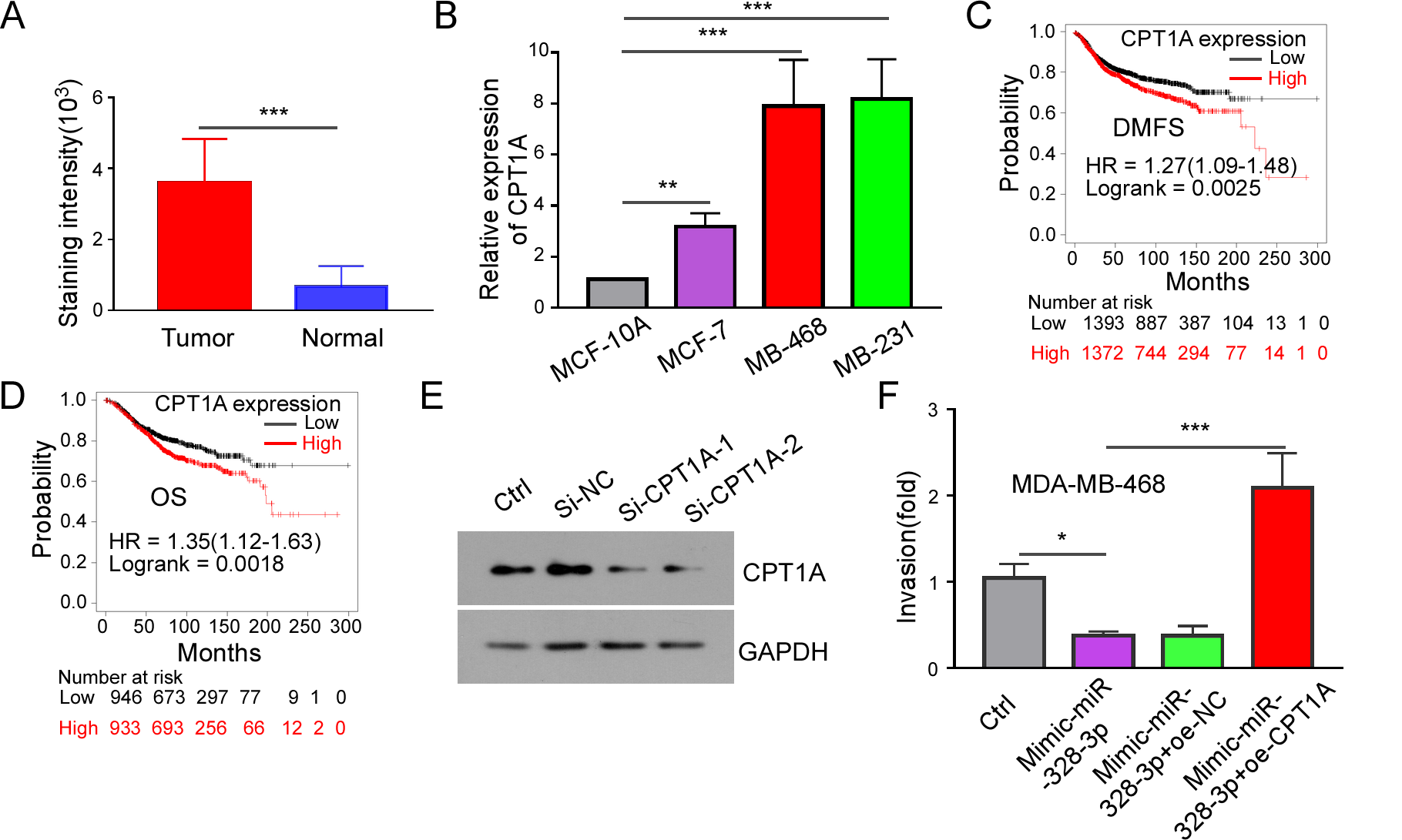


**Supplementary Data 3: CPT1A regulates breast cancer metastasis**

**A,** Quantitative analysis of immunohistochemistry results in Fig. 4B (n = 55; ****p* < 0.001 by student’s t test).

**B,** The relative expression of CPT1A in MCF-10A, MCF-7, MDA-MB-468 and MDA-MB-231 cells was detected by RT-qPCR. Mean ± SD, ***p* < 0.01, ****p* < 0.001 compared to MCF-10A by student’s t test (n = 3).

**C and D,** Kaplan-Meier Plotter analysis of distant metastasis-free survival (DMFS) and overall survival (OS) of breast cancer was shown.

**E,** The CPT1A inhibition efficiency via si-RNA was detected by immunoblot in MDA-MB-231.

**F,** The invasion capability of MDA-MB-468 transfected with mimic-miR-328-3p with or without CPT1A overexpression was detected by Transwell assay. Mean ± SD, **p* < 0.05, ****p* < 0.001 compared to 2^nd^ group by One-Way ANOVA with Dunnett-t test (n = 3).


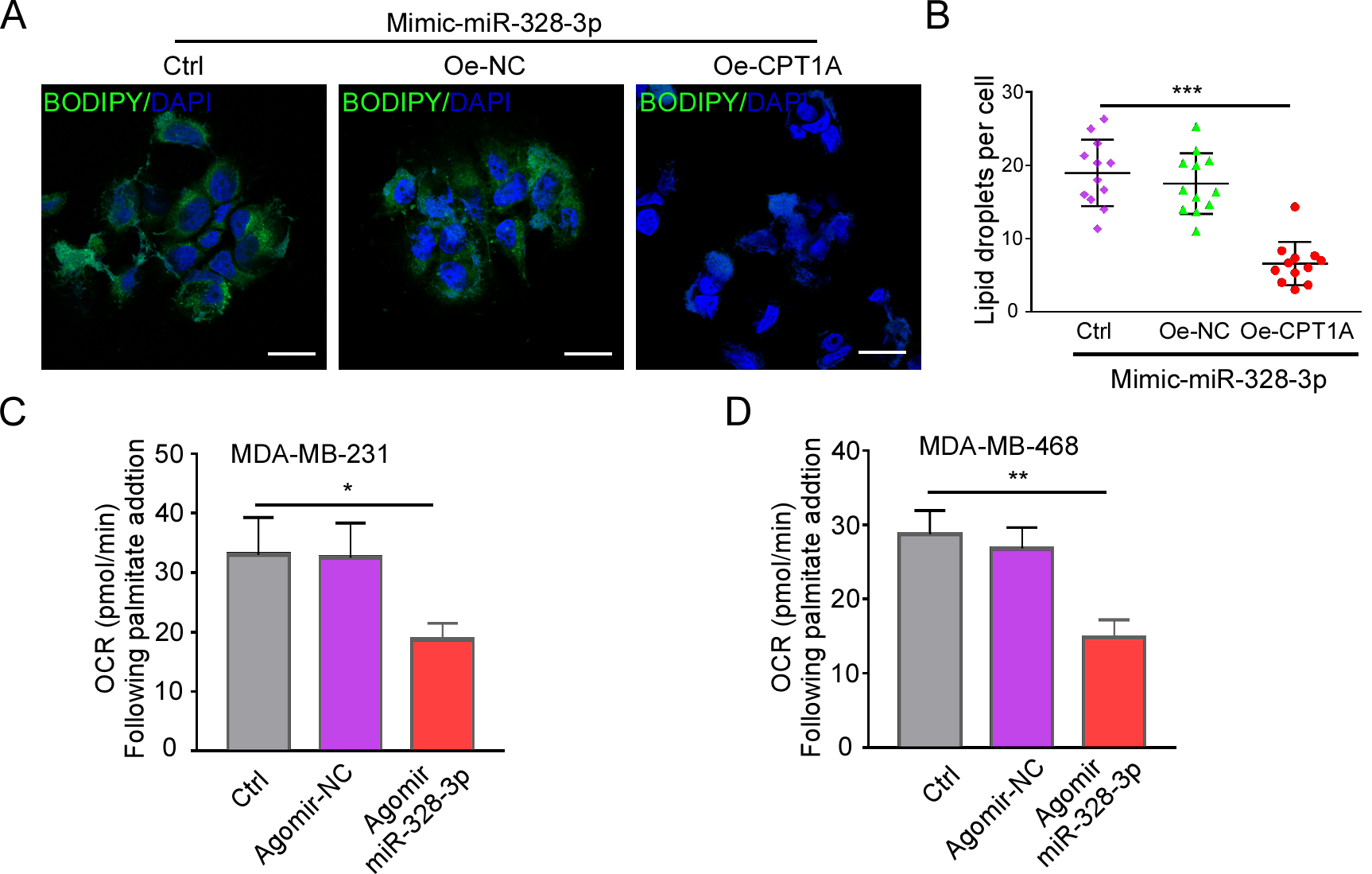


**Supplementary Data 4: MiR-328-3p controls fatty acid β-oxidation by CPT1A**

**A and B,** The lipid droplets level in MDA-MB-468 cells transfected with mimic-miR-328-3p with or without CPT1A overexpression was detected by BODIPY 493/503 staining. Representative image (A) and fluorescence foci quantification (B) are shown. Scale bar: 20 μm. Mean ± SD, ****p* < 0.001 by One-Way ANOVA with Dunnett-t test , n = 12 in 3 independent experiment.

**C and D,** Changing in OCR (ΔOCR) follow palmitate-BSA addition was calculated as (OCR at the time of palmitate-BSA injection—final basal OCR) in MDA-MB-231 (C) and MDA-MB-468 (D) with Agomir miR-328-3P. Mean ± SD, **p* < 0.05, ***p* < 0.01 by One-Way ANOVA with Dunnett-t test (n = 3).


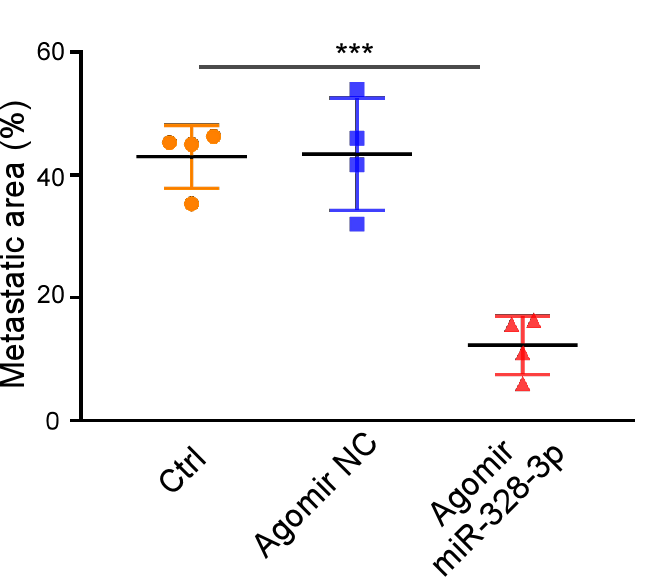


**Supplementary Data 5: MiR-328-3p inhibits the metastasis of breast cancer.** Quantitative analysis of the percentage lung metastatic area in Fig. 6F (n = 4 mice/group; ****p* < 0.001 by one-way ANOVA with Dunnett-t test).


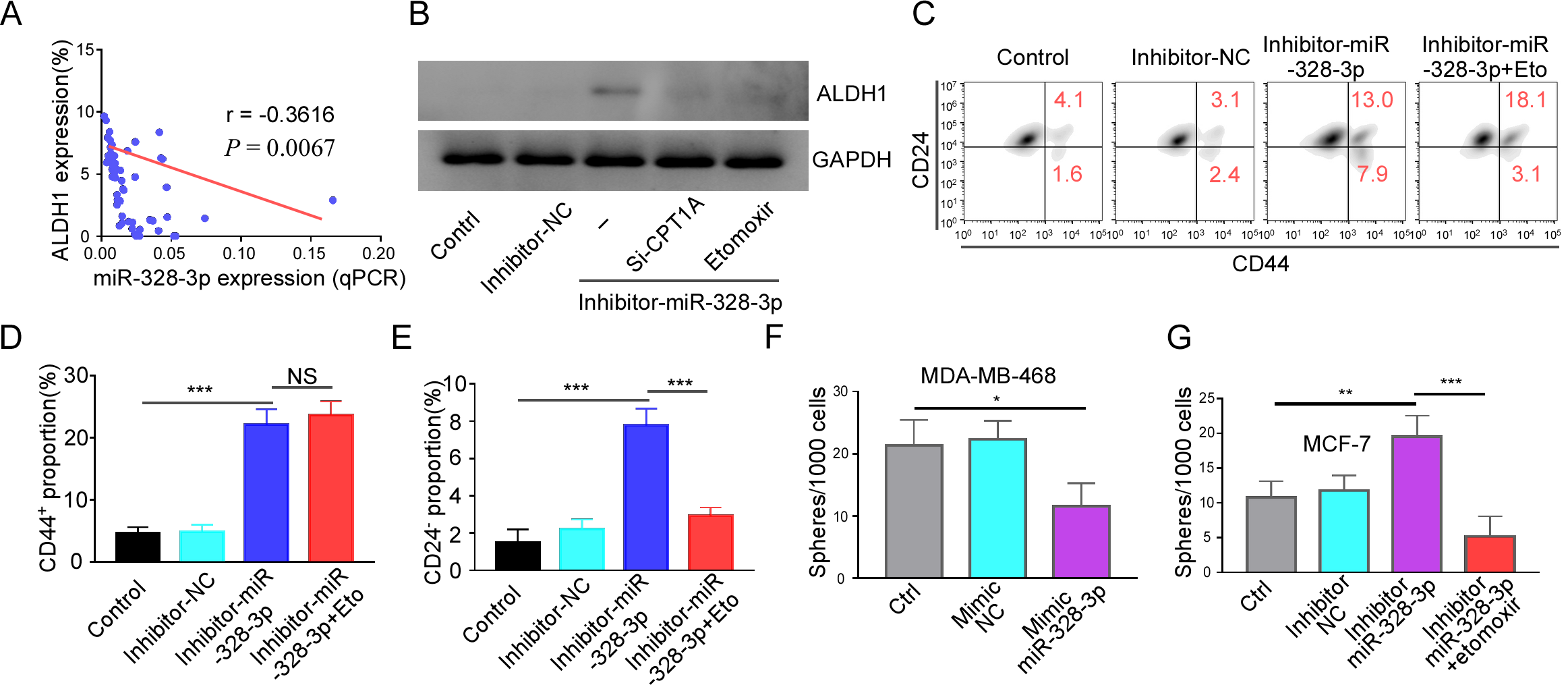


**Supplementary Data 6: MiR‑328-3p depletion enhances cancer cells stemness**

**A,** ALDH1 and miR-328-3p were detected by confocal and RT-qPCR, respectively, in 55 breast cancer patients, the relation of ALDH1 and miR-328-3p was shown.

**B,** The ALDH1 expression in MCF-7 cells treated with inhibitor-miR-328-3p and FAO inhibitor, silenced CPT1A or etomoxir, was checked by western blot, n = 3.

**C-E,** CD44^+^ and CD24^-^ proportion in MCF-7 cells treated with inhibitor-miR-328-3p and etomoxir (Eto) were checked by flow cytometry (C), D and E is statistic graph of C, NS represents not significant, ****p* < 0.001 by one-way ANOVA with Dunnett-t test, n = 3.

**F,** Quantification of sphere formation in Fig. 7D. Mean ± SD, **p* < 0.05 by One-Way ANOVA with Dunnett-t test (n = 3).

**G,** Quantification of sphere formation in Fig. 7E. Mean ± SD, ***p* < 0.01, ****p* < 0.001 by One-Way ANOVA with Dunnett-t test (n = 3).
