# Supplementary figures and images for: Fatty acid β-oxidation promotes breast cancer stemness and metastasis via the miRNA-328-3p-CPT1A pathway
Source: Cancer Gene Ther. 2021 May 27;29(3-4):383–95. doi: 10.1038/s41417-021-00348-y (PMC8940624; doi:10.1038/s41417-021-00348-y)

# Fig. 3E

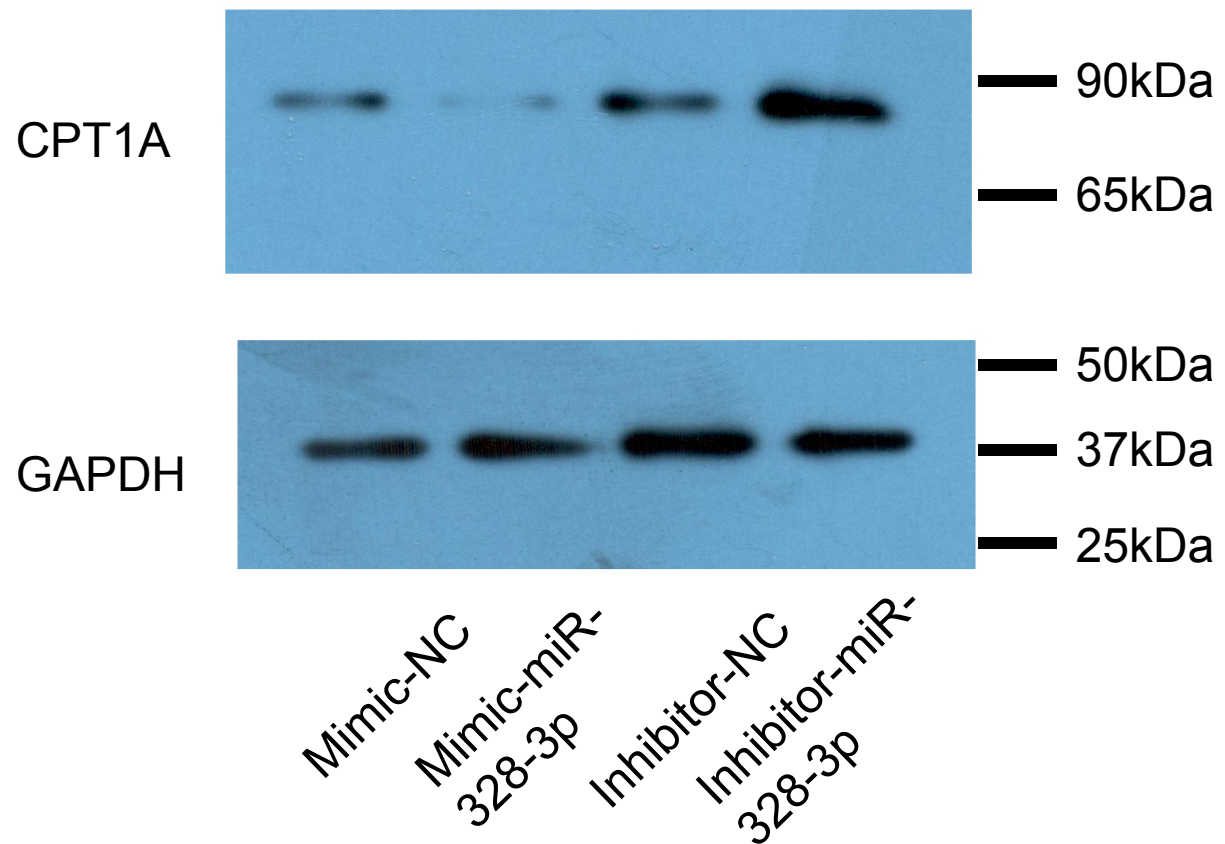

# Fig. 3G

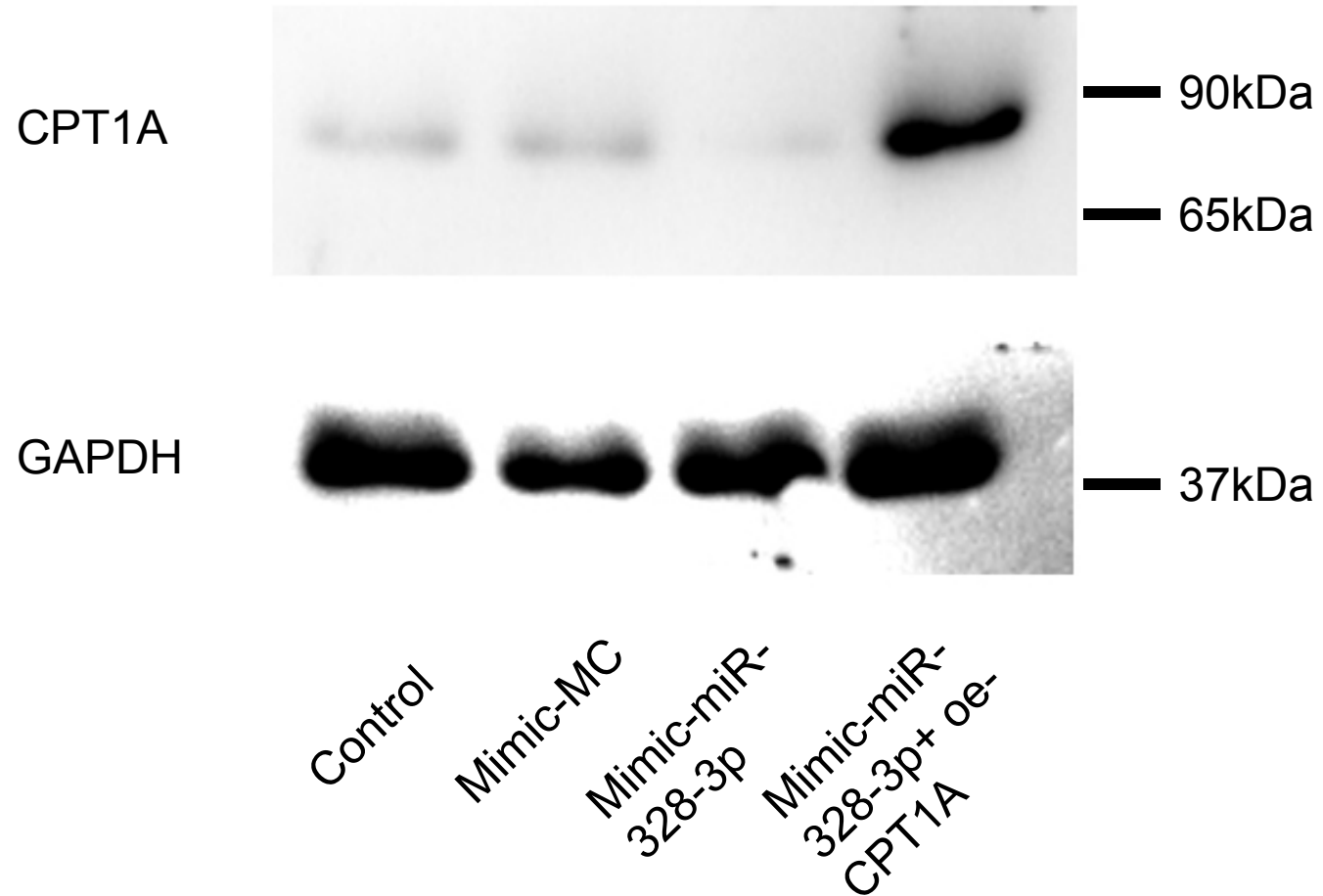

# Fig. 6C

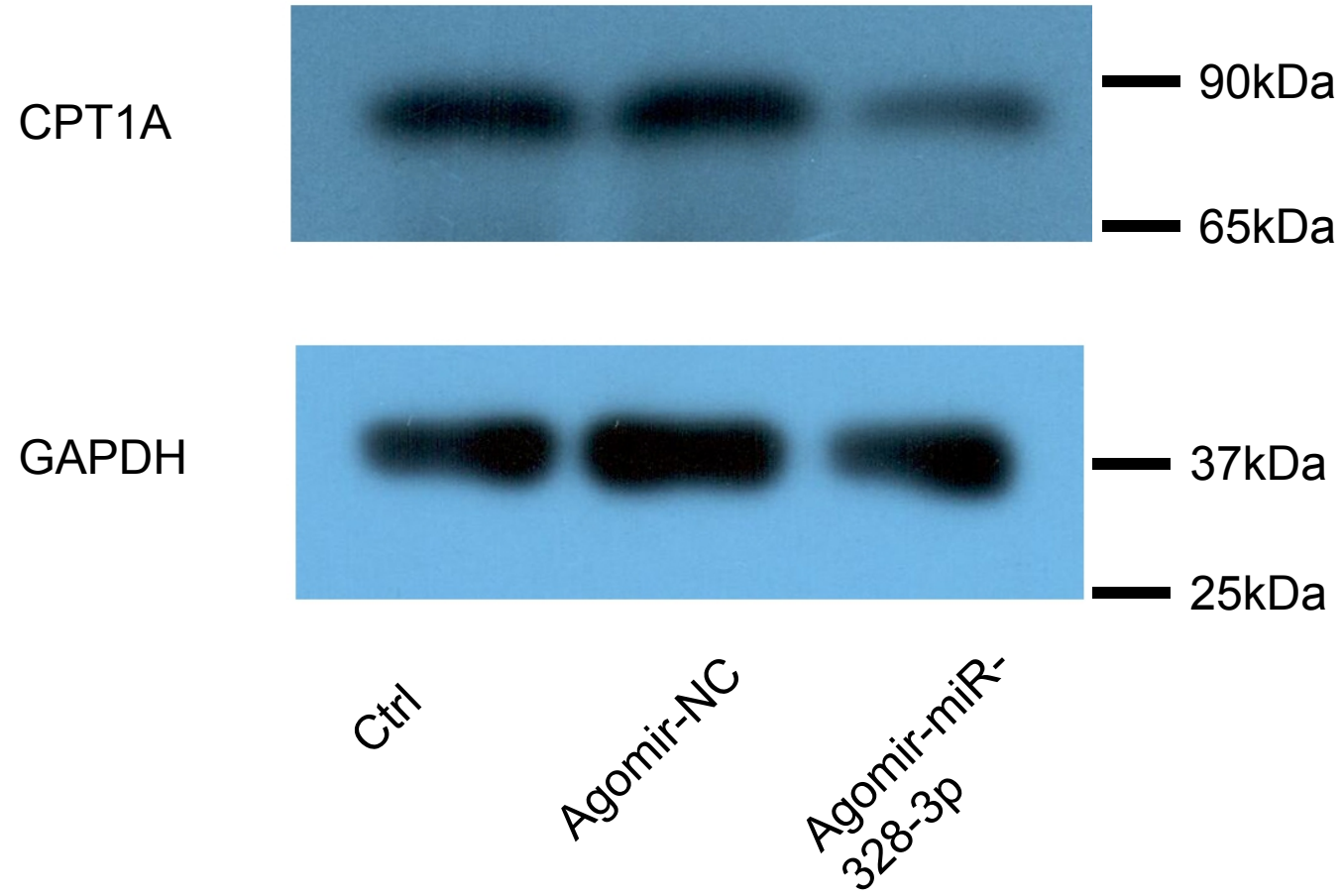

# Supplementary Data 3E

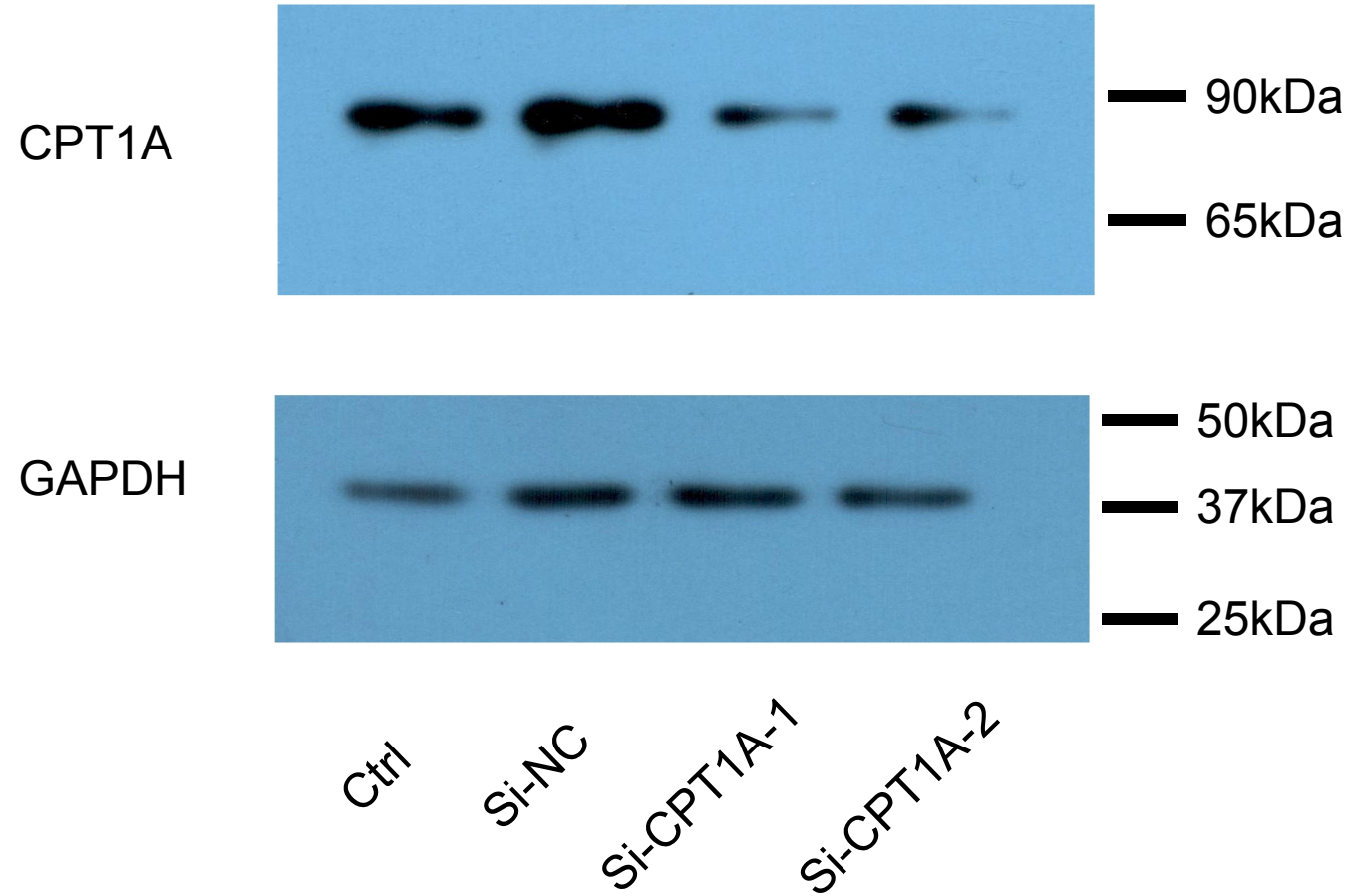

# Supplementary Data 6B

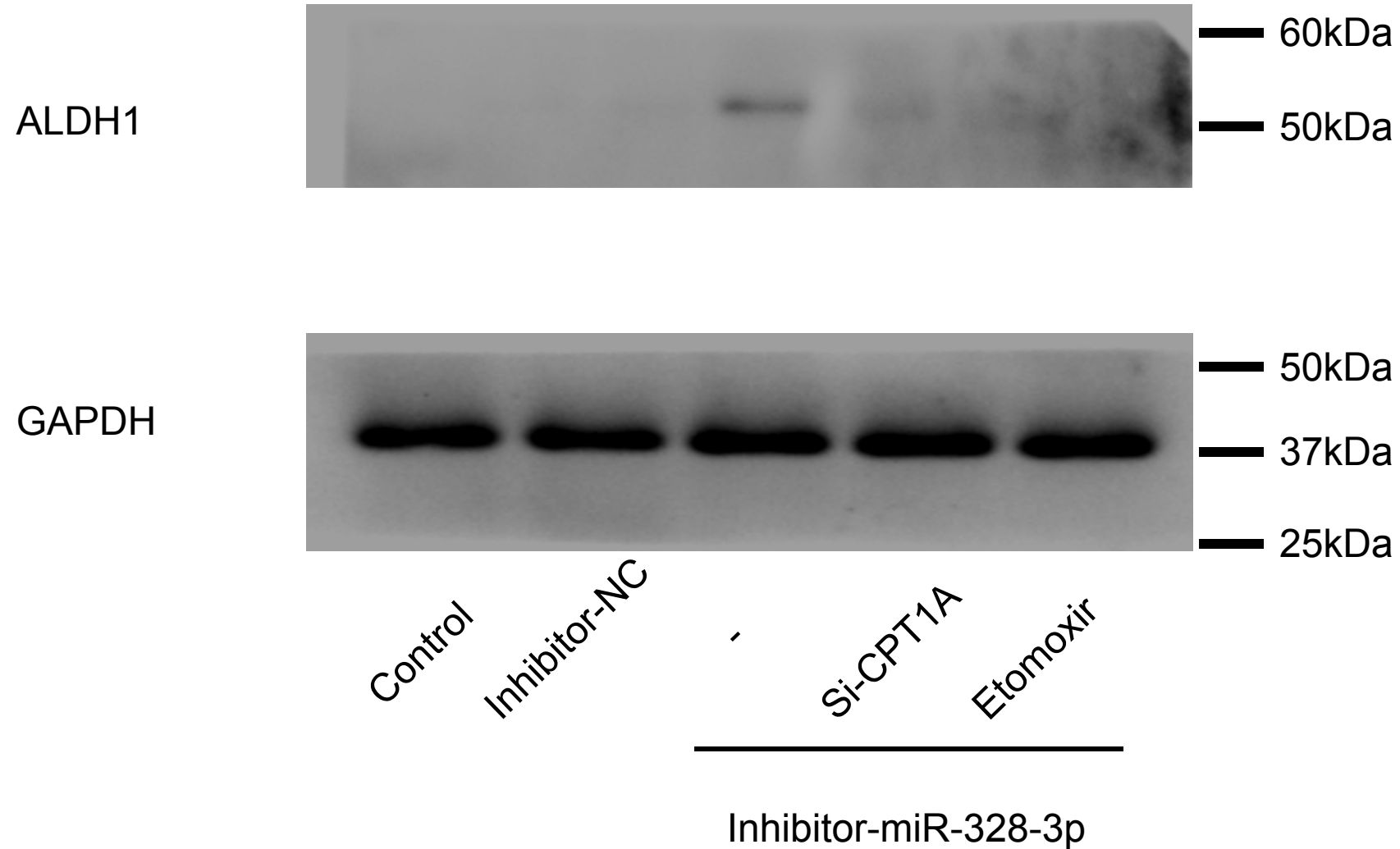

Supplement: Supplementary file 2 — primary WB [file 41417_2021_348_MOESM2_ESM.pdf]
